# Editorial: Identification and functional dissection of stress-responsive genes in cotton

**DOI:** 10.3389/fpls.2022.1008064

**Published:** 2022-10-18

**Authors:** Jiahe Wu, Peng Wang, Xiaoyang Ge

**Affiliations:** ^1^ The State Key Laboratory of Plant Genomics, Institute of Microbiology, Chinese Academy of Sciences, Beijing, China; ^2^ State Key Laboratory of Cotton Biology, Institute of Cotton Research of Chinese Academy of Agricultural Sciences, Anyang, China

**Keywords:** cotton, stress, *verticillium dahliae*, drought, salt, resistance

This editorial provides a summarization of the contributions to the Frontiers Research Topic “*Identification and Functional Dissection of Stress-responsive Genes in Cotton*”, identifying many important genes related to biotic and abiotic stress-response in cotton and dissecting their function mechanism.

Cotton is an important cash crop that provides a good natural fiber and feed oil worldwide. The cultural cotton species include *Gossypium hirsutum*, *G. barbadense*, *G. herbaceum*, and *G. arboreum*, of which the first two species are generally planted in China. However, cotton production is limited by various biotic and abiotic stresses, which lead to loss in cotton yield and quality. Therefore, we conducted this Research Topic to promote cotton studies on levels of plant resistance to various stresses.

In this Research Topic, a total of 25 papers have been published: 24 original research papers and one review. Some of the papers conducted similar research models, in which authors first identified a gene family, and then chose candidate genes to dissect their function in plant defense *via* ectopic expression in *Arabidopsis thaliana* and knockdown of gene expression by the virus-induced gene silencing (VIGS) method. Other papers firstly identified defense-related genes and clarified their functions in plant resistance biotic and abiotic stresses *via* genetic materials. And the defense mechanism underlying these genes were dissected by physiological, biochemical, and histological methods. These defense genes could be regarded as candidate genes to accelerate cotton defense breeding. We shall provide a summarization of these papers.

## Identification and dissection of plant resistance genes to *V. dahliae* infection

Cotton verticillium wilt, a notorious disease mainly caused by *V. dahliae* infection, damages cotton production due to loss of fiber yield and quality. Therefore, identification of disease-resistant genes is important to breed resistant cotton cultivars to control this wilt disease damage. In the Research Topic, there were eight papers focusing on disease-resistant gene identification and function dissection. In three papers, authors identified the disease-resistant gene families and dissected the function mechanism of candidate genes. Wu et al. identified 96, 44, and 57 CDPKs (calcium-dependent protein kinases) from *G. hirsutum*, *G. raimondii*, and *G. arboretum*, respectively. Phosphoproteomics analysis showed that *V. dahliae*-induced GhCDPK28-6 localized in the cell membrane was phosphorylated under the stress of *V. dahliae* and may interact with GhPBL9 and GhRPL12C. Knockdown of *GhCDPK28-6* leads to an increase in reactive oxygen species and plant resistance, while the overexpression of *GhCDPK28-6* in *A. thaliana* weakened the plant resistance to the pathogen. Li et al. identified 141 CC-NBS-LRR (CNL) genes from *G. barbadense*. Knockdown of *GhCNL130* compromised plant resistance to *V. dahlae*, whereas ectopic overexpression of this gene in *Arabidopsis* could increase plant resistance. Ren et al. showed data reporting 24 cotton SEVEN IN ABSENTIA (SINA) ubiquitin ligases, among which *GhSINA7*, *GhSINA8*, and *GhSINA9* were upregulating expression at 24 h after inoculation with *V. dahliae*. *In vitro* ubiquitination assays indicated that the three GhSINAs possess E3 ubiquitin ligase activities, and they could interact with each other *via* yeast two-hybrid screening. The ectopic overexpression of *GhSINA7*, *GhSINA8*, or *GhSINA9* in *A. thaliana* resulted in increased tolerance to *V. dahliae*, while individual knockdowns of *GhSINA7*, *GhSINA8*, and *GhSINA9* compromised it. The other three papers revealed that three miRNA-targeted gene modules function in fine-tuning plant resistance against *V. dahliae* infection. Wei et al. revealed that GhLAC4 participates in lignin biosynthesis and plant resistance against *V. dahliae* under the regulation of ghr-miR397. *GhLAC4* knockdown and ghr-miR397 overexpression significantly reduced basal lignin content and major G-lignin content. Under *V. dahliae* infection, G-lignin content in ghr-miR397-knockdown plants significantly increased. The extract-free stems of various genetic plants lost significantly different weights when treated with commercial cellulase and *V. dahliae* secretion compared to the control, suggesting that lignin protects plant cell walls from degradation mediated by cellulase or fungal secretions. Jia et al. revealed that cotton Teosinte branched1/Cincinnata/Proliferating cell factor (TCP) 4-like transcription factor participates in plant defense *via* regulation of miR319b, which could interact with NON-EXPRESSER OF PATHOGEN-RELATED GENES 1 (NPR1) to directly activate isochorismate synthase 1 (*ICS1*) expression to promote salicylic acid (SA) accumulation. These results were confirmed by silencing of ghr-miR319b and GhTCP4-like plants. Shi et al. characterized *GhTIR1* as being targeted by ghr-miR393 in plant defense. GhTIR1 interacts with GhIAA14 to affect plant resistance to *V. dahliae* infection. And GhTIR1 functions in plant resistance involving SA accumulation. Overall, the ghr-miR393-GhTIR1 module participates in plant response to *V. dahliae* infection *via* IAA perception and signaling partially depending on the SA defense pathway. Additionally, Sun et al. revealed cotton SWEET (sugars will eventually be exported transporter) protein 42 can be induced upon *V. dahliae* infection in roots. Overexpression of *GhSWEET42* in *Arabidopsis* decreased plant resistance against *V. dahliae*, whereas knockdown of *GhSWEET42* in cotton increased plant resistance. Cui et al. identified two QTLs related to verticillium wilt resistance, qvw-D05-1 and qvw-D05-2, *via* BSA-seq analysis using an F2:3 segregation population. RNA-seq analysis revealed that *GhDRP* in qvw-D05-2 can be remarkably induced by *V. dahliae* infection. However, the knockdown of *GhDRP* decreased plant resistance to this fungus.

## Identification and dissection of abiotic stress-responding genes

Although cotton has a higher tolerance to abiotic stress compared to other crops, it is not able to grow in extreme environmental conditions. Therefore, it is important to milt defense genes to various abiotic stresses, including drought, salt, cold, etc.

## Identification and dissection of drought-tolerant genes

In this Research Topic, four papers demonstrated several key genes participating in plant tolerance to drought stress. Mahmood et al. used ten cotton genotypes, three drought-tolerant and seven susceptible, to identify drought-related genes *via* F1 cross combinations along with 10 parents under non-stress and 50% drought stress treatments to assess the effects of drought stress and its inheritance in the next generation. The results revealed that days taken to maturity, yield traits, and physiological traits are under significant genetic control for improving drought tolerance. Mehari et al. identified 13 hub genes related to the drought stress response. Among these, Gh_A06G1257 had the highest expression under drought stress. Overexpression of Gh_A06G1257 in *Arabidopsis* increased plant drought tolerance, confirmed in the knockdown of Gh_A06G1257 cotton plants. Bano et al. screened four cultural *Gossypium* species for tubby-like proteins (TLPs) proteins to obtain 105 members. Cotton TLP gene family members expanded mainly due to segmental duplication. Among these, *GhTLP11A* and *GhTLP12A.1* genes function in salt and drought stress responses. Zhang et al. identified 18 HD-ZIP III genes from *G. hirsutum*, among which GhHB8-5D is targeted to the cell nucleus and has self-activation ability. The ectopic expression of *GhHB8-5D* or its synonymous mutant *GhHB8-5Dm* in *Arabidopsis* resulted in the changes in vascular bundles and deposition of secondary walls, leading to a significant increase in plant drought tolerance.

## Identification and dissection of salt-tolerant genes

There are five papers that identified salt-tolerant genes from cotton and revealed the mechanism underlying their function. Sun et al identified 156 plant cytochrome P450 (P450) in *G. hirsutum*, which were divided into four subfamilies. Most of the P450 were likely to be involved in the salt tolerance of cotton seedlings. Lu et al. identified the ethylene response factor (ERF) subfamily B3 group from 4 *Gossypium* spp. And GhERF13.12 was confirmed to participate in plant salt tolerance. *GhERF13.12* transgenic *Arabidopsis* showed enhanced salt tolerance and the silencing of the *GhERF13.12* led to increased sensitivity to salt stress in cotton. Liu et al. cloned a chloride channel gene (GhCLCg-1) from upland cotton localized on the vacuolar membrane, responding to chloride stress (NaCl or KCl). The overexpression of *GhCLCg-1* in *A. thaliana* decreased the Na^+^/K^+^ ratios of absorption to enhance salt tolerance. In contrast, silencing *GhCLCg-1* in cotton plants increased the Cl^–^ contents and the Na^+^/K^+^ ratios, resulting in compromised salt tolerance. Zhan et al. performed miRNAs, mRNA, and degradome sequencing and combined the three omics data to analyze the salt-response genes. 399 candidate targets of salt-induced miRNAs were identified, and 72 targets of 25 miRNAs were verified by degradome sequencing data. Among these, miR390 and miR393 overexpression increased plant sensitivity to salt stress. Zhang et al. identified 44 KNOX (KNOTTED1-like homeobox) transcription factors from *G. hirsutum*. Among these KNOXs, silencing of *GhKNOX2* enhanced the salt tolerance of cotton seedlings, whereas silencing of *GhKNOX10* and *GhKNOX14* reduced seedling tolerance to salt stress.

## Identification and dissection of cold-tolerant and insect pest-resistant genes

Two interesting papers showed that several important genes participate in plant tolerance to cold stress and plant resistance to insect pests. Chen et al. sought membrane attack complex/perforin (MACPF) genes in four cotton species and acquired 100 members. Cotton MACPF gene family members expanded mainly through the whole-genome duplication (WGD)/segmental. In addition, silencing *GhMACPF26* was shown to increase cold tolerance. Zhang et al. carried out the transcriptome profiles between resistant and susceptible cotton cultivars under whitefly infestation. A total of 606 differentially expressed lncRNAs were identified, among which lncA07 and lncD09 were potential hub genes that play a regulatory role in cotton defense against aphid infestation. CRISPR/Cas9 knock-out mutant of *lncD09* and *lncA07* showed a decrease in jasmonic acid (JA) content, leading to increased susceptibility toward insect infestation.

Additionally, there are four papers that identified dual- or multi-role genes in plant tolerance to drought and salt stresses. Fu et al. identified 56 caleosin (CLO) protein from cotton species that might be regulated by abscisic acid and methyl jasmonate. Transcriptome data revealed that *GhCLO* genes responded to salt and drought stresses. *GhCLO06*-silencing reduced plant salt tolerance, involving the upregulation of ABA-related genes, *GhABF2*, *GhABI5*, and *GhNAC4*. Bano et al. exploited a transcriptomics meta-analysis to identify the candidate genes related to response to drought and salt stresses. Several hub genes, including *NSP2*, *DRE1D*, and *ERF61*, were identified to participate in plant tolerance to drought and salt stresses. Liu et al. employed the BC2F2 population among *G. tomentosum* and *G. hirsutum* as wild male donor parents noted for its drought tolerance to identify drought tolerance genes *via* genotyping by sequencing. Golden2-like (GLK) gene, belonging to the GARP transcription factor family, is characterized as a drought and cold response gene. GLK knockdown reduced cotton plant drought and cold tolerance, whereas its overexpression in *Arabidopsis* can increase plant drought and cold tolerance. Li et al. obtained 17 cotton fructose-1,6-biphosphate aldolase (*FBA*) genes *via* screening *G. hirsutum* genome. Transcriptome and qRT-PCR analysis showed that most *GhFBAs* could respond to various abiotic stress and phytohormonal treatments.

There is a review in this Research Topic that helps us to understand key genes and mechanisms in cotton defense against biotic and abiotic stresses. And this review provided a good statement on the regulatory network of cotton genes in response to salt, drought, and wilt diseases as well as a statement of progress and perspective (Billah et al.).

There is also a paper that studies plant growth and development. Li et al. identified 39 EXORDIUM (EXO) genes as the indicators of BR response genes from *G. hirsutum*. *GhEXO7_At* was mainly expressed in response to BL treatment. And *GhEXO7_At* ectopic expression in *Arabidopsis* and silencing affected plant growth and development involved in the BR signaling pathway. It is notable that plant defense against stresses is closely related to plant growth and development.

## Author contributions

JW was one of the guest associate editors of the Research Topic and wrote the paper. XG played important roles in handling the manuscripts of the Research Topic and summarized the publications. PW edited the text and added links to papers. All authors contributed to the article and approved the submitted version.

## Funding

This work was supported by the National Natural Science Foundation of China (31971905) and was sponsored by the State Key Laboratory of Cotton Biology Open Fund (CB2021B02).

## Acknowledgments

Our guest editor group, Jiahe Wu, Hong Zhang, Qing Liu, Lanqin Xia, Jinfa Zhang, and Fuguang Li, thank the authors of the papers published on this Research Topic for their valuable contributions and the referees for their rigorous review. We also thank the editorial board of the Plant Abiotic Stress section for their support.

## Conflict of interest

The authors declare that the research was conducted in the absence of any commercial or financial relationships that could be construed as a potential conflict of interest.

## Publisher’s note

All claims expressed in this article are solely those of the authors and do not necessarily represent those of their affiliated organizations, or those of the publisher, the editors and the reviewers. Any product that may be evaluated in this article, or claim that may be made by its manufacturer, is not guaranteed or endorsed by the publisher.

